# APR-246 reactivates mutant p53 by targeting cysteines 124 and 277

**DOI:** 10.1038/s41419-018-0463-7

**Published:** 2018-04-18

**Authors:** Qiang Zhang, Vladimir J. N. Bykov, Klas G. Wiman, Joanna Zawacka-Pankau

**Affiliations:** 1 Department of Oncology and Pathology, Cancer Center Karolinska (CCK), Karolinska Institutet, SE-17176 Stockholm, Sweden; 2 Department of Microbiology, Tumor and Cell Biology (MTC), Karolinska Institutet, SE-17177 Stockholm, Sweden

## Abstract

The *TP*53 tumor suppressor gene is frequently inactivated in human tumors by missense mutations in the DNA binding domain. *TP*53 mutations lead to protein unfolding, decreased thermostability and loss of DNA binding and transcription factor function. Pharmacological targeting of mutant p53 to restore its tumor suppressor function is a promising strategy for cancer therapy. The mutant p53 reactivating compound APR-246 (PRIMA-1^Met^) has been successfully tested in a phase I/IIa clinical trial. APR-246 is converted to the reactive electrophile methylene quinuclidinone (MQ), which binds covalently to p53 core domain. We identified cysteine 277 as a prime binding target for MQ in p53. Cys277 is also essential for MQ-mediated thermostabilization of wild-type, R175H and R273H mutant p53, while both Cys124 and Cys277 are required for APR-246-mediated functional restoration of R175H mutant p53 in living tumor cells. These findings may open opportunities for rational design of novel mutant p53-targeting compounds.

## Introduction

Tumor suppressor p53 is a transcription factor that responds to cellular stress, e.g., DNA damage and oncogene activation. p53 triggers cell cycle arrest, senescence and apoptosis^1,2^. More recent studies have shown that p53 also has roles in metabolism^3^, stem cell division^4^, fertility^5^ and cell death by ferroptosis^6^. The *TP*53 gene is inactivated in a large fraction of human tumors^7,8^. The majority of *TP*53 mutations are missense mutations resulting in substitution of amino acid residues that make direct contact with DNA, such as R248W and R273H, or residues that are important for the structural integrity of the core domain, e.g. R175H and R249S. This leads to loss of specific DNA binding^9^.

The high frequency of *TP*53 mutations in human tumors has stimulated efforts to develop therapeutic strategies for targeting mutant p53 in cancer. Several low molecular weight compounds have been reported to restore wild-type function to mutant p53, including PRIMA-1 and the PRIMA-1 analog APR-246 (PRIMA-1^Met^)^10–18^. In addition to targeting mutant p53, APR-246 inhibits the selenoprotein thioredoxin reductase 1 (TrxR1) and converts it to an active oxidase^19^, and depletes cellular gluthatione (GSH)^20–22^. This induces reactive oxygen species (ROS) and presumably contributes to the anticancer effect of APR-246^23^. Liu et al. showed that *SLC7A11* expression is a new marker for sensitivity of mutant p53-carrying tumors to APR-246/MQ. Mutant p53 binds to the antioxidant transcription factor Nrf2, leading to decreased expression of *SLC7A11*, which sensitizes cells to TrxR1 inhibition and GSH depletion by APR-246/MQ^22^. APR-246 has been tested in a phase I/IIa clinical trial in patients with hematological malignancies or prostate cancer^24^. APR-246 is converted to methylene quinuclidinone (MQ), a Michael acceptor that reacts with cysteines in the p53 core domain^25^. However, the mechanism by which APR-246/MQ reactivates mutant p53 is not fully understood.

Many mutant p53 proteins are thermodynamically unstable at body temperature^26^. Studies of temperature-sensitive mutants suggest that stabilization of conformation is critical for regaining wild-type p53 activity^27,28^. Thus, pharmacological stabilization of mutant p53 should allow its functional rescue and efficient elimination of tumor cells^29^.

Here we examined the role of the Michael acceptor activity of MQ for thermostabilization of wild-type (wt) and mutant p53 core domains and refolding of R175H mutant p53 in living cells. We also show that Cys277 is essential for MQ-mediated thermostabilization of R175H and R273H mutant p53 core domains, and that both Cys124 and Cys277 are required for APR-246-mediated R175H mutant p53 reactivation in tumor cells.

## Results

### MQ binds to the p53 core domain via Michael addition

**Fig. 1:**
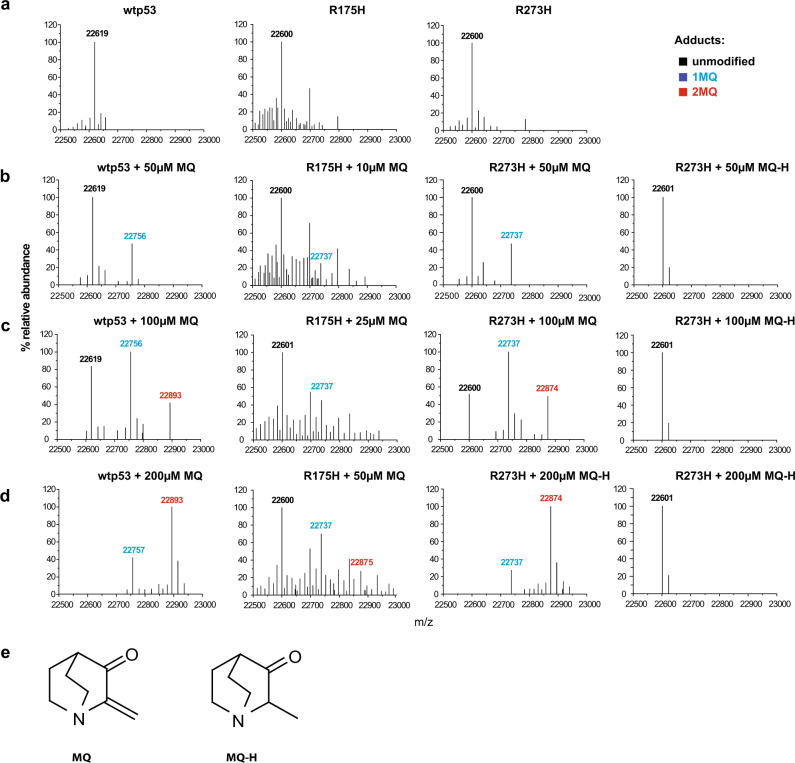
MQ binds to cysteine residues in wild-type and mutant p53 core domains in a dose-dependent manner. Mass measurement of wild-type, R273H and R175H p53 core domains by LTQ-MS. **a** mass spectra of p53 core domains. **b**–**d** Reaction titration with MQ or MQ-H. p53 core domains were incubated with MQ at 50–200 µM (wt and R273H) or 10–50 µM (R175H) concentration ranges. One MQ adduct increased the molecular mass of p53 core domains by 137 Da. **e** Structure of MQ and MQ-H.

First, we studied MQ binding to p53 by Nanomate Linear Ion Trap Orbitrap hybrid mass spectrometry (LTQ-MS). Accurate masses of the p53 core domains are shown in Fig. 1a. We incubated wt and R273H mutant p53 core domains (20 µM) with increasing concentrations of MQ at 21 °C and assessed MQ adduct formation by LTQ-MS. The deconvoluted mass spectra showed that 32% of the wt and R273H p53 core domain proteins were modified by one MQ molecule when incubated with lower concentrations of MQ (Fig. 1b), and that all p53 protein molecules had two MQ adducts upon incubation with 200 µM MQ (Fig. 1d). We did not detect modification of all 10 cysteines in the p53 core at these concentrations of MQ.

Due to low yield of the R175H p53 core domain, we analyzed it at lower protein concentration. LTQ-MS indicated that 20% of the R175H core was modified by one MQ molecule at 10 µM (Fig. 1c), and at 50 µM MQ, 35% of the R175H core domain protein had one MQ adduct and 14% had two MQ adducts (Fig. 1d). Thus, the number of MQ-modified cysteine residues increased in a concentration-dependent manner.

Next, we assessed the binding of MQ-H, a hydrogenated analog of MQ that lacks reactive carbon-carbon double bond (Fig. 1e). We did not detect any modification at concentrations up to 200 µM, indicating that MQ-H does not modify cysteine residues in p53. Thus, the Michael acceptor activity of MQ is required for modification of cysteines in p53.

### MQ enhances thermostability of the p53 core domain

**Fig. 2:**
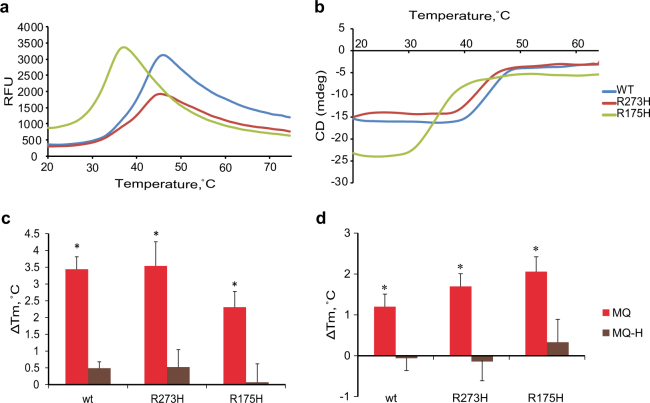
MQ modification of cysteine residues enhances p53 core domain thermostability. Melting curves of wt and R273H and R175H mutant p53 core domains as assessed by DSF (**a**) and CD at 218 nm (**b**). Changes in *T*_m_ after MQ or MQ-H modification as assessed by DSF (**c**) and CD at 218 nm (**d**) (mean ± SD, *n* = 3). All proteins were thermostabilized by MQ modification (red bars), but not by MQ-H (brown bars). * p < 0.05 (student *t*-test).

To test if MQ modification of cysteines increases thermostability of the p53 core domain, we applied differential scanning fluorimetry (DSF). This allows analysis of the interactions between a protein and a ligand based on changes in the protein melting temperature (*T*_m_). DSF (Fig. 2a) demonstrates that the wt p53 core domain was the most stable of the three proteins with a *T*_m_ of 40.38 ± 0.06 °C. *T*_m_ for the DNA contact mutant R273H was 39.16 ± 0.52 °C, whereas the structural mutant R175H was the least stable protein with a *T*_m_ of 30.64 ± 0.46 °C, consistent with previously published studies^26^. To further validate this result, we used circular dichroism spectroscopy (CD), which allows assessment of α-helix and β-sheet structure content. We performed CD analysis at 218 nm since both *α*-helix and *β*-sheet structures are detected at this wavelength. In agreement with our DSF data, CD (Fig. 2b) measurements demonstrated that the wt p53 core domain is the most stable protein followed by the R273H core domain whereas the R175H core is considerably less stable. The *T*_m_ values are 44.74 ± 0.08 °C, 43.96 ± 0.37 °C and 36.24 ± 0.33 °C, respectively.

We then incubated wt, R273H and R175H p53 core domains with 2 mM MQ and performed DSF and CD analyses. This concentration of MQ was chosen to override the reducing agents DTT or TCEP included in the reaction buffer to preserve the structure of the p53 core domains. According to DSF, MQ increased the *T*m value of wt, R273H and R175H p53 core domains by 3.44, 3.54 and 2.31 °C, respectively (Fig. 2c). This degree of thermal stabilization by small molecules is in agreement with previously published results^16^. CD analysis confirmed thermostabilization of all three cores by MQ as shown by the increase in *T*_m_ values by 1.20, 1.70, and 2.06 °C for wt, R273H and R175H p53, respectively (Fig. 2d). The inactive MQ analog MQ-H did not significantly change the thermal stability of the p53 core domains (Fig. 2c, d).

Thus, MQ but not MQ-H increases the thermostability of p53 core domains, confirming that cysteine binding by Michael addition is critical for p53 thermostabilization by MQ. The results from DSF and CD were fully consistent; the protein melting temperatures determined by the two methods correlated with each other (*r* = 0.999, *p* = 0.007). DSF was chosen for further studies.

### Identification of MQ binding sites in the p53 core domain

The p53 core domain has 10 cysteine residues with varying solvent accessibility. Previous studies have indicated that in the absence of DNA, Cys277 has the highest solvent accessibility, followed by Cys182 and Cys229, whereas Cys135, Cys141 and Cys275 have poor solvent accessibility^30^. Cys124 is located at the center of the flexible L1/S3 pocket, which can be stabilized by second-site mutations to rescue mutant p53 folding^31–33^. Interestingly, Cys124 shows a nuclear magnetic resonance (NMR) chemical shift upon binding of the CDB3 peptide that stabilizes mutant p53^34^. In addition, substitution of Cys124 abrogated reactivation of R175H mutant p53 by PRIMA-1^31^.

**Fig. 3:**
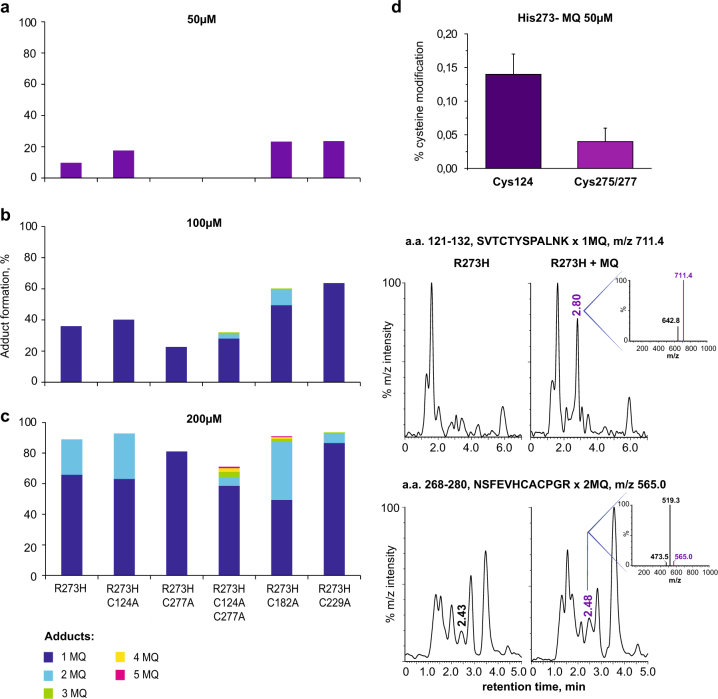
Cys277 is a prime binding site for MQ in the p53 core domain. Mass measurement of p53 core domains by LTQ-MS. **a**–**c** Indicated p53 core domain proteins were incubated with MQ at increasing concentrations and mass shift was assessed. Proportion of MQ-modified proteins was calculated based on relative intensity of each detected mass. **d** Modification of Cys124 and Cys275/277 in the R273H mutant p53 core domain after treatment with 50 µM MQ as assessed by LC-MS after tryptic digestion of MQ-treated protein (mean ± SE, *n* = 3).

To investigate if the most solvent-exposed cysteine residues and Cys124 are critical for MQ binding to p53, we introduced single Cys to Ala substitutions at position 124, 182, 229 or 277 in the wt, R175H and R273H p53 core domains, and double substitutions at Cys124 and Cys277 in the same three core domains. The R175H p53 core domain has low intrinsic thermostability and additional amino acid substitutions might destabilize it further. This probably explains why we obtained negligible protein yield for the R175H–C124A and R175H–C124A–C277A core domains. LTQ-MS analysis of the R273H mutant p53 core domains identified one MQ adduct in the R273H, R273H–C124A, R273H–C182A, and R273H–C229A p53 cores after incubation with 50 µM MQ, whereas no MQ modification of the R273H–C277A and R273H–C124A–C277A core domains was detected (Fig. 3a). However, incubation with 100–200 µM MQ resulted in 2–5 MQ adducts in all p53 mutant core domains tested except R273H–C277A (Fig. 3b, c). This implies that MQ modifies several cysteines in p53, and suggests that Cys277 is the most reactive.

To confirm modification of Cys124 and Cys277 by MQ, we incubated recombinant R273H mutant p53 core domain with 50 µM MQ, digested with trypsin, and analyzed peptides by LC-MS (liquid chromatography-mass spectrometry). We detected modification of p53 peptides SVTCTYSPALNK and NSFEVHVCACPGR that contain Cys124 and Cys275/277, respectively. The degree of modification of Cys124 and Cys275/277 could be estimated to 0.14 ± 0.03% and 0.04 ± 0.02 (mean ± SE, *n* = 3), respectively. Amount of each peptide is expressed as a percentage of the amount of the corresponding unmodified peptide detected in the same sample according to LC-MS (Fig. 3d).

### Cys277 is essential for MQ-mediated thermostabilization of p53 core domains

**Fig. 4:**
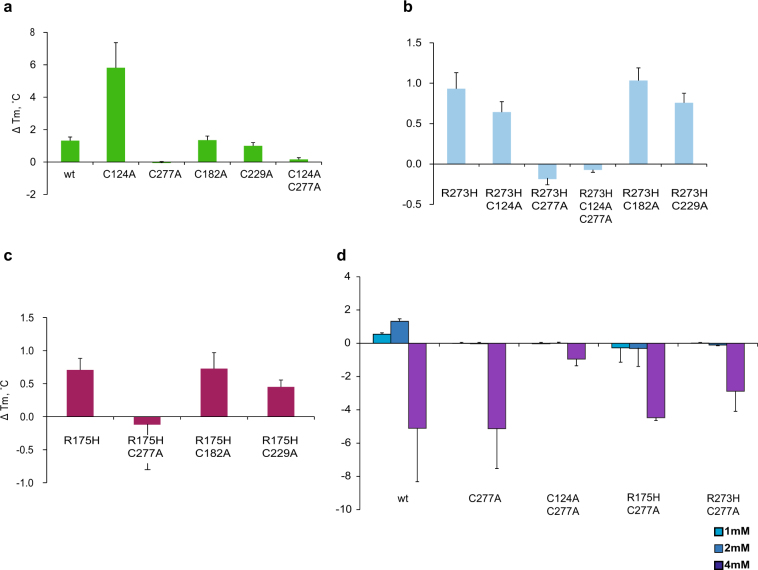
Cys277 is critical for MQ-mediated p53 thermostabilization. C277A substitution completely abolished MQ-mediated thermostabilization whereas other substitutions had little or no effect on wt **a**, R273H **b** and R175H **c** core domains at 2 mM concentration. Higher concentrations of MQ (1, 2 or 4 mM) did not thermostabilize the indicated p53 core domain proteins with C277A substitution **d**.

To assess the role of selected Cys residues in MQ-mediated thermostabilization, we incubated wt, R175H and R273H p53 core domains carrying indicated Cys to Ala substitutions with MQ and performed DSF. The *T*_m_ values of the wt and p53 core domains carrying C124A, C277A, C182A, C229A, and C124A–C277A substitutions were 42.36, 36.67, 43.07, 42.43, 40.59 and 40.91 °C, respectively (Table 1). MQ modification increased the *T*_m_ values of the C124A, C182A, and C229A p53 core domains by 5.82, 1.35 and 1.00 °C (Fig. 4a), respectively. In contrast, MQ caused a slight destabilization of the C277A core domain by −0.06 °C, and only stabilized the C124A–C277A core domain by 0.16 °C, indicating that Cys277 is critical for MQ-mediated thermostabilization of p53.

**Table 1:** Melting temperatures (*T*_m_) of p53 core domain proteins with indicated Cys to Ala substitutions as assessed by DSF^a^

*T*_m_ (°C)	wt	C124A	C277A	C182A	C229A	C124A–C277A
wt	42.36 ± 0.11	36.67 ± 1.29	43.07 ± 0.26	42.43 ± 0.12	40.59 ± 0.12	40.91 ± 0.26
R175H	31.09 ± 0.23	—	29.05 ± 0.50	29.49 ± 0.37	28.16 ± 0.46	—
R273H	40.49 ± 0.93	39.17 ± 0.08	41.48 ± 0.23	40.52 ± 0.06	38.60 ± 0.08	39.48 ± 0.10

The differences in the *T*_m_ values of wt, R175H and R273H p53 core domains between Table 1 and data presented in the main text (Results p. 5–6) are due to different batches of protein and iCycler instruments for DSF

^a^ Sufficient amounts of the R175H–C124A and R175H–C124A–C277A mutant proteins could not be obtained for DSF analysis

We then analyzed R273H p53 core domains with the same C124A, C277A, C182A and C229A substitutions. The melting temperatures of the R273H, R273H–C124A, R273H–C227A, R273H–C182A, and R273H–C229A p53 core domains were 40.49, 39.17, 41.48, 40.52, and 38.60 °C, respectively (Table 1). MQ shifted the *T*_m_ values by 0.93, 0.64, −0.19, 1.03 and 0.76 °C (Fig. 4b), respectively. The *T*_m_ of the R273H core domain with C124A–C277A double substitution was 39.48 °C and only changed by −0.07 °C upon MQ modification.

As indicated above, we were not able to obtain sufficient amounts of the R175H–C124A and R175H–C124A–C277A p53 core domains. Thus, only the R175H, R175H–C277A, R175H–C182A and R175H–C229A core domains were further analyzed. Their respective melting temperatures were 31.09, 29.05, 29.49, and 28.16 °C (Table 1). MQ modification shifted the melting temperatures by 0.71, −0.12, 0.73, and 0.45 °C (Fig. 4c), respectively. Thus, C227A substitution abrogated MQ-induced thermostabilization in wt and R273H and R175H mutant p53 core domain backgrounds.

Our MS data demonstrate that MQ binds to the p53 core domain in a concentration-dependent manner and that high concentrations of MQ lead to modification of more than one cysteine residue. This raises the question whether high concentrations of MQ might induce p53 thermostabilization even in the absence of Cys277. To address this, we incubated wt, C277A, C124A–C277A, R175H–C277A and R273H–C277A p53 core domains with 1, 2 or 4 mM MQ and performed DSF (Fig. 4d). The highest concentration of MQ (4 mM) destabilized all core domains (Fig. 4d). The wt p53 core was stabilized by MQ at 1 and 2 mM, as observed previously. However, p53 core domains with the C277A substitution were not stabilized at the same concentrations. This further supports our conclusion that Cys277 is indispensable for MQ-mediated p53 core domain stabilization.

### APR-246 and MQ induce R175H mutant p53 refolding to wild-type conformation

Proper folding of p53 is crucial for sequence-specific DNA binding and transactivation of p53 target genes. Most *TP*53 mutations lead to unfolding and loss of sequence-specific DNA binding. PRIMA-1 was shown to promote R175H mutant p53 refolding to wild-type conformation in SKOV-His175 cells^11^. However, this has yet to be demonstrated for APR-246 and MQ.

The monoclonal antibody PAb1620 detects properly folded p53 but not unfolded p53. We verified the specificity of this antibody in immunostaining by treating HCT116 human colon carcinoma cells with doxorubicin to induce the levels of wild-type p53. HCT116 cells expressing wild-type p53 or Saos-2 cells expressing R273H mutant p53, which retains wild-type-like conformation, showed positive PAb1620 staining. In contrast, H1299 cells expressing R175H mutant p53 were PAb1620 negative. Treatment with APR-246 induced positive PAb1620 staining in H1299–R175H cells, suggesting that APR-246 via MQ restores wild-type conformation of this mutant (Supplementary Figure 1).

**Fig. 5:**
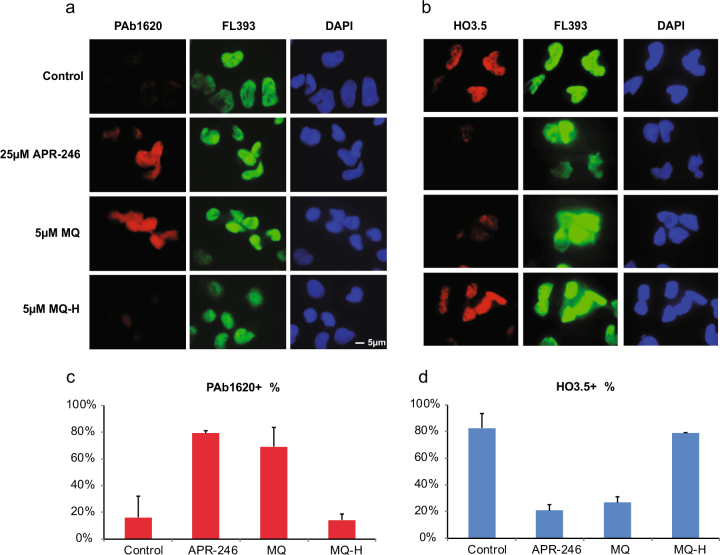
APR-246 and MQ enhance wild-type p53 conformation-specific PAb1620 epitope in tumor cells carrying R175H mutant p53. **a** Immunofluorescence staining of TOV-112D cells treated with APR-246, MQ or MQ-H using wild-type p53 conformation-specific antibody PAb1620 and co-immunostaining with general p53 antibody FL-393. **b** Immunofluorescence staining of TOV-112D cells treated with APR-246, MQ or MQ-H using the mutant p53 conformation-specific antibody HO3.5 and co-immunostaining with general p53 antibody FL-393. **c** Quantification of PAb1620 immunostaining. **d** Quantification of HO3.5 immunostaining. PAb1620 or HO3.5-positive cells were counted in 15 fields from 3 independent experiments. The number of PAb1620 or HO3.5-positive cells was divided by the total cell number to obtain percentage of positive cells.

To determine if APR-246 and MQ can refold endogenous R175H mutant p53 in TOV-112D ovarian carcinoma cells, we treated the cells with APR-246 or MQ and performed co-immunostaining with PAb1620 and anti-p53 polyclonal antibody FL-393. In parallel, the cells were stained with the monoclonal HO3.5 antibody that specifically detects unfolded p53 similarly to the PAb240 antibody^35^. APR-246 treatment increased PAb1620 staining (Fig. 5a, c) and decreased HO3.5 staining (Fig. 5b, d), indicating refolding of R175H mutant p53 to wild-type-like conformation.

Next, we treated TOV-112D cells with MQ and stained with PAb1620 and HO3.5 antibodies. Like APR-246, MQ-induced PAb1620 staining (Fig. 5a), which coincided with decreased HO3.5 staining (Fig. 5b). We did not detect any changes in PAb1620 and HO3.5 staining after treatment with MQ-H, confirming that the Michael acceptor activity of MQ is crucial for mutant p53 refolding in living tumor cells.

### Cysteines 124 and 277 are important for APR-246/MQ-mediated R175H mutant p53 reactivation

**Fig. 6:**
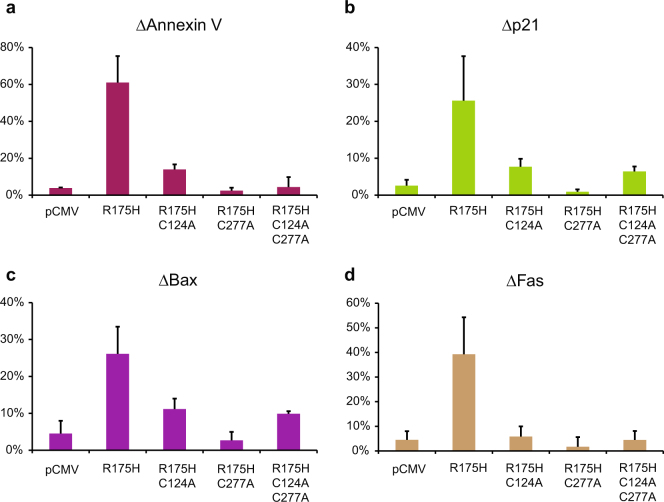
Cys124 and Cys277 are crucial for APR-246/MQ-mediated R175H reactivation in living cells. H1299 cells expressing corresponding p53 mutant proteins were stained with Annexin V, p21, Fas and Bax and examined by flow cytometry. Both C124A and C277A abolished APR-246-induced apoptosis **a**, and upregulation of p53 targets p21 **b**, Bax **c** and Fas **d**

To investigate the role of cysteine residues in mutant p53 reactivation by APR-246/MQ in tumor cells, we transiently transfected p53 null H1299 cells with vectors encoding R175H, R175H–C124A, R175H–C277A or R175H–C124A–C277A p53 mutant proteins, or with control vector (pCMV). Western blotting confirmed similar levels of p53 expression in all transfectants (Supplementary Figure 2). Next, the cells were treated with 45 µM APR-246. We chose this concentration since it induced cell death in R175H mutant p53-transfected cells but only marginally affected empty vector-transfected cells. We then assessed apoptosis and expression of p53 targets p21, Fas and Bax by flow cytometry. Signals obtained in mock-treated control cells were subtracted from the signals obtained in APR-246-treated cells, and the values are presented as relative increase (ΔAnnexin V, Δp21, ΔFas, and ΔBax). Cells transfected with R175H showed substantial induction (61.06%) of Annexin V staining after APR-246 treatment when compared to cells transfected with empty vector (3.91%). We observed a relatively small induction of Annexin V staining by APR-246 in cells transfected with R175H–C124A p53 (13.96%), and cells expressing R175H–C277A or R175H–C124A–C277A p53 showed no induction of Annexin V staining as compared to empty vector-transfected cells (Fig. 6a). The p53 targets p21 (Fig. 6b) and Bax (Fig. 6c) were highly induced by APR-246 in cells expressing R175H p53 but only slightly or moderately increased in cells expressing R175H–C124A and R175H–C124A–C277A mutants. Cells expressing R175H–C277A mutant p53 showed no induction of p21 or Bax. The p53 target Fas (Fig. 6d) was strongly induced by APR-246 in R175H-transfected cells but no induction compared to control-transfected cells was detected in cells expressing R175H–C124A, R175H–C277A or R175H–C124A–C277A p53 proteins, consistent with the observed absence of apoptosis induction.

To determine whether the C124A and C277A substitutions themselves affect wild-type p53 function, H1299 cells transfected with wt, C124A, C277A, R175H and R273H p53 constructs were assessed for apoptosis and expression of p53 target p21 by flow cytometry. pCMV vector was used as a control. Induction of Annexin V (Supplementary Figure 3a) was detected in cells expressing wt, C124A or C277A p53 proteins, which coincided with slight induction of p21 (Supplementary Figure 3b), but not in cells transfected with R175H or R273H p53 constructs. Thus, C124A or C277A substitution *per se* does not inactivate p53 in this experimental system, supporting our conclusion that these cysteines play a key role in APR-246/MQ-mediated mutant p53 reactivation.

## Discussion

Small molecules that reactivate mutant p53 by restoring wild-type conformation have been identified by various approaches^10,23,36^. APR-246 has been tested in a phase I/IIa clinical trial in patients with hematological malignancies or prostate cancer^24,37^, and is currently tested in a phase II trial in patients with high-grade serous (HGS) ovarian cancer (http://www.clinicaltrials.gov/ct2/show/NCT02098343). We previously demonstrated thiol modifications in the p53 core domain by PRIMA-1 conversion products^25^. This led us to conclude that APR-246-mediated mutant p53 reactivation involves covalent binding of MQ. Other mutant p53-reactivating compounds, such as MIRA-1^38^, CP-31398 and STIMA-1^39^, 3-benzoylacrylic acid^14^, 2-sulfonylpyrimidines^16^, and the curcumin analog HO-3867^18^ possess similar thiol reactivity, indicating that the observed association between thiol reactivity and mutant p53 reactivation is not coincidental.

Here we show that the MQ analog MQ-H that lacks a reactive carbon-carbon double bond and therefore lacks Michael acceptor activity, does not modify cysteine residues in the p53 core domain, does not enhance p53 thermostability and does not induce R175H mutant p53 refolding according to PAb1620 staining. Thus, by using several approaches, we demonstrate that the electrophilic properties of MQ are essential for cysteine modification, thermostabilization and refolding of mutant p53.

Although our previous studies indicated that PRIMA-1 conversion products bind covalently to the p53 core domain^25^, the exact cysteine targets for MQ have remained unknown. We applied LTQ-MS analysis to a set of Cys to Ala mutants to identify cysteine residues that are critical for MQ binding and MQ-mediated stabilization of mutant p53. The reactivity of cysteine residues in a protein is largely affected by their solvent accessibility. Among 10 cysteines in p53 core domain, Cys176, Cys238, and Cys242 coordinate a zinc ion which is responsible for holding p53 loops together^9^, making them less likely targets for modification. Cys135, Cys141, and Cys275 are poorly accessible to solvent based on the X-ray crystal structure of the p53 core domain. Cys277 and Cys182 have the highest solvent accessibility, followed by Cys229^30^. Interestingly, Cys277 has the lowest p*K*_a_ of all p53 cysteines, making it the strongest nucleophile in the protein. Thus, Cys277 combines the greatest solvent accessibility with the highest nucleophilicity, suggesting that it might be a prime target for MQ.

Indeed, we found that Cys277 to Ala substitution abolishes MQ binding to p53 core domain, at lower concentrations. Moreover, the ability of MQ to thermostabilize p53 core domains is impaired in Cys277 to Ala mutants. Thus, a good correlation exists between the extent of MQ adduct formation and MQ-mediated thermostabilization of the p53 core domain.

Cys182 and Cys277 have recently been identified as prime binding sites for PK11000, a 2-sulfonylpyrimidine compound that reacts with cysteines and thermostabilizes p53^16^. It is noteworthy that although Cys277 interacts directly with DNA, modification of this residue by PK11000 did not change p53 DNA binding and transactivation of target genes. The mutant p53-targeting curcumin analog HO-3867 also binds covalently to Cys182 and Cys277^18^. However, our results demonstrate that substitution of Cys182 to Ala does not affect p53 modification and thermostabilization by MQ in any significant way.

Kaar and colleagues^14^ identified 3-benzoylacrylic acid as a thiol-binding compound that reacts first with Cys124 and Cys141 and to a lesser extent with Cys135, Cys182 and Cys277 in p53. Cys124 was also identified as a target for PRIMA-1 by molecular modeling, and Cys124 to Ala substitution abolished PRIMA-1-induced reactivation of mutant p53 in human tumor cells^31^. Here we found that substitution of this cysteine did not impair MQ binding and p53 thermostabilization. However, Cys124 to Ala substitution abrogated R175H reactivation by APR-246/MQ in tumor cells, in agreement with the results of Wassmann et al.^31^.

It is unclear why the C124A mutant core domain was more efficiently stabilized by MQ than wt p53 or R273H–C124A p53 (Fig. 4). Notably, Cys124 to Ala substitution decreases p53 core domain stability more extensively in a wt background. Although the structures of wt and R273H p53 core domains are presumably similar, it is difficult to predict local structural changes after introduction of C124A. Thus, this question can probably only be resolved by NMR and/or X-ray crystallography analysis of MQ-modified p53.

To exclude the possibility that the Cys to Ala substitutions themselves impair wild-type p53 function in our experimental setting, we examined whether the Cys to Ala substitutions affect the ability of p53 to transactivate p21 and induce apoptosis. We confirmed that these substitutions do not affect normal p53 function to any major extent, supporting the notion that the observed effects are indeed due to an important role of Cys124 and Cys277 for APR-246/MQ-mediated mutant p53 reactivation.

In conclusion, our data demonstrate that specific cysteines are critical targets for mutant p53 reactivation by APR-246/MQ. Our findings may open opportunities for designing novel compounds targeting mutant p53 based on a similar mechanism of nucleophilic addition at the identified binding sites.

## Materials and Methods

### Cell lines and reagents

Human lung adenocarcinoma cells H1299 and osteosarcoma cells Saos-2 are p53 null. The sub-lines H1299–R175H and Saos-2-R273H stably express the indicated mutants^11,38^. Human HCT116 colon carcinoma cells express wild-type p53. Human epithelial ovarian cancer cells TOV-112D express R175H mutant p53. All cells were cultured at 37 °C, 5% CO_2_ in IMDM medium (Hyclone, Logan, Utah) supplemented with 10% FBS (Thermo Fisher Scientific, Waltham, MA).

APR-246, MQ, and MQ-H were obtained from Aprea Therapeutics AB, Stockholm, Sweden. Methanol, formaldehyde, and acetonitrile were purchased from Thermo Fisher Scientific (Waltham, MA). Formic acid was purchased from Sigma-Aldrich (St. Louis, MO). Lipofectamine 2000 was from Thermo Fisher Scientific (Waltham, MA). All solvents were of analytical grade and are commercially available.

Rabbit polyclonal anti-p53 FL-393, rabbit polyclonal anti-GAPDH, mouse monoclonal anti-p53 DO-1 and mouse monoclonal PAb1620, Alexa Fluor 647 conjugated FL-393, Alexa Fluor 488 conjugated anti-p21 antibodies were from Santa Cruz Biotechnology (Heidelberg, Germany). Mouse monoclonal antibody HO3.5 was a gift from Professor Thierry Soussi, Karolinska Institutet. Polyclonal rabbit anti-Bax Biotin OAAF02999 and Qdot^TM^ 605 streptavidin were from Nordic Biosite (Stockholm, Sweden). BV510 mouse anti-human CD95 (Fas) and BD Horizon V450 Annexin V were from BD Biosciences (Stockhlom, Sweden).

### Site-directed mutagenesis

Prokaryotic and eukaryotic plasmid constructs were produced by Genscript, Piscataway, NJ.

### Expression and purification of proteins

p53 cores (94–292) were cloned into pNIC28-Bsa4 that adds an N-terminal hexahistidine tag and transformed into *E. coli* strain Rosetta2 (DE3). Bacteria were grown in TB medium supplemented with 8 g/l glycerol at 37 °C with shaking. Protein expression was induced with 0.5 mM IPTG at 18 °C overnight. Afterwards bacteria were pelleted by centrifugation and lyzed in cold IMAC lysis buffer (50 mM TRIS, 300 mM NaCl, 10% glycerol, 0.05 mM ZnCl, 0.5 mM TCEP, pH 8.0) supplemented with complete protease mix (complete EDTA-free (protease inhibitor) and 5 μl benzonase nuclease (250 U) and stored at −80 °C. After thawing, the cells were lyzed by pulsed sonication (4 s/4 s 3 min, 80% amplitude), centrifuged (20 min at 49,000×*g*) and the soluble fractions were decanted and filtered through 0.45μm filters. The samples were loaded onto the ÄKTA Xpress LC and purified overnight. His-tag was cleaved with Thrombin. Sample homogeneity was confirmed by mass spectrometry and the concentration was measured by nanodrop. The proteins were aliquoted and stored at −80 °C in storage buffer (50 mM TRIS, 800 mM NaCl, 10% glycerol, 2.0 mM TCEP, pH 8.0).

### Mass spectrometry

Wild-type and R273H p53 core domains were de-salted against 20 mM ammonium acetate buffer by using 10 K concentration columns (Vivaspin, GE Healthacare, Chicago, IL). Twenty µM of the purified protein were incubated with 0 µM (control), 50, 100 or 200 µM MQ for 15 min at 21 °C. R175H core domains were de-salted by ZipTip C4 resin tips for MALDI-ToF MS (Merck Millipore, Billerica, MA) following the manufacturer’s protocol. 3.2 µM of R175H protein were treated with 0 µM (control), 10, 25 or 50 µM of MQ for 15 min at 21 °C. 5% formic acid (1:1 volume ratio) was added to the samples to increase the ionization sensitivity. Samples were analyzed by LTQ XL mass spectrometry (Thermo Fisher Scientific, Waltham, MA) fitted with an automated nanospray source (TriVersa Nanomate, Advion Biosciences, Ithaca, NY) using nanoelectrospray chips with spraying nozzels. The ion source was controlled using the Chipsoft 8.3.1 software (Advion Biosciences, Ithaca NY). Three microliters of each sample were loaded into a 96-well plate and injection volume was one and a half microliters. Full scan spectra were collected at the m/z 500–2000 in positive ion mode. The mass spectra of each sample were acquired in profile mode over 4 min. The spectra were analyzed using XCalibur^TM^ Software (Thermo Fisher Scientific, Waltham, MA). Deconvoluted ESI spectra are presented.

### LC-MS

30 μg of R273H p53 core domain protein was treated with 50 μM MQ in 20 mM ammonium bicarbonate pH 8.0 for 1 h at room temperature. Samples were then precipitated with acetone and pellets were digested with trypsin at 37 °C for 3 h. 10 µl of each sample was injected onto Waters Alliance HPLC system (Waters, Sollentuna, Sweden) and resolved on XSelect^®^ Peptide CHSTM C18, XP column, 130 Å, 2.5 μm, 2.1 × 150 mm (Waters, Sollentuna, Sweden). The peptide separation was performed by water-acetonitrile gradient elution supplemented with 0.1% formic acid. Peptides were detected with Acquity QDa MS detector in ESI + mode. Data were analyzed by MassLynx software (Waters, Sollentuna, Sweden).

### Circular dichroism

75 μg p53 core domain proteins were incubated with or without 2 mM MQ in 250 µl 40 mM potassium phosphate buffer (pH 7.5) and 1 mM DTT for 1 h at 21 °C. The concentration of p53 was 13.3 μM. CD measurements were performed on Jasco-810 (Jasco Inc., Tokyo, Japan) with 1.00 mm pathlength. Denaturation curves were obtained by measuring the CD spectra at 218 nm. Melting temperatures were analyzed by GraphPad Prism 6 (Graphpad Software Inc, La Jolla, CA) according to the Boltzmann equation $y = \frac{{A_1 - A_2}}{{1 + e^{\left( {x - x_0} \right)/dx}}} + A_2$, *x*_o_ - inflection point.

### Differential scanning fluorimetry

5 μg p53 core domain proteins were incubated with 1–4 mM MQ in 25 μl 40 mM potassium phosphate buffer (pH 7.5) with 1 mM DTT for 1 h at 21 °C under controlled conditions. The concentration of p53 was 8.8 μM. 1 μl of 25× Sypro orange were added to each well. The fluorescence was assessed by Bio-Rad iCycler (Bio-Rad Laboratories, CA) at increasing temperature from 10 to 75 °C with a rate of 1 °C per min. Tm values were calculated by GraphPad Prism 6 (Graphpad Software Inc, La Jolla, CA) in the same way as for CD.

### Immunofluorescence staining

Cells were plated into a 16-well chamber slide at a density of 3000 cells per well, allowed to attach overnight, and treated with 25 µM APR-246 or 5 µM MQ/MQ-H for 16 h. Cells were washed, fixed with 4% formaldehyde and permealized with 0.2% Triton X. Mouse PAb1620 or HO3.5 antibody were co-incubated with rabbit FL-393 antibody, all were diluted 1:200 in 2% BSA for 1 h at 4 °C. Anti-rabbit Alexa 488 and anti-mouse Alexa 594 conjugates were used as secondary antibody with 1:200 dilution in 2% BSA.

### Flow cytometry

Cells were grown on 6-well plates at an initial density of 500,000 cells/well. Sixteen hours later cells were transfected for 24 h with p53 expression vectors or empty vector using Lipofectamine 2000 according to the manufacturer’s protocol (Life Technology, Waltham, MA). The medium was then replaced with fresh medium, the cells were reseeded at a density of 20,000 cells/well after 6 h culture, and treated with APR-246 on the following day. Cells were collected 24 h post-treatment, stained with Annexin V, fixed with 4% formaldehyde, permealized with 90% methanol and stained with Fas, Bax, and p21 antibodies. Cells were analyzed on a NovoCyte Flow Cytometer (ACEA Biosciences, Solna, Sweden).

## Electronic supplementary material

Supplementary Figure 1(PDF 2454 kb)

Supplementary Figure 2(PDF 427 kb)

Supplementary Figure 3(PDF 7 kb)

Supplementary information(DOCX 16 kb)
