# Correction: APR-246 reactivates mutant p53 by targeting cysteines 124 and 277

**DOI:** 10.1038/s41419-019-1997-z

**Published:** 2019-10-10

**Authors:** Qiang Zhang, Vladimir J. N. Bykov, Klas G. Wiman, Joanna Zawacka-Pankau

**Affiliations:** 1 Department of Oncology and Pathology, Cancer Center Karolinska (CCK), Karolinska Institutet, SE-17176 Stockholm, Sweden; 2 Department of Microbiology, Tumor and Cell Biology (MTC), Karolinska Institutet, SE-17177 Stockholm, Sweden

**Keywords:** Allergy, Acid, base, fluid, electrolyte disorders, Acute coronary syndromes, Asthma, Acid, base, fluid, electrolyte disorders, Astronomy and astrophysics, Bone, Acid, base, fluid, electrolyte disorders, Biliary tract, Bioremediation, Cell biology, Cell growth, Acute coronary syndromes

**Correction to: Cell Death and Disease**

10.1038/s41419-018-0463-7, published online 18 April 2018

Since publication of this article, the authors have noticed that there was an error in Fig. 1d, third panel from left, “R273H + 200 μM MQ-H” should be “R273H + 200 μM MQ”. A corrected version of Fig. 1 is included here. Our corrections do not affect the original conclusions of this paper. The authors would like to apologize for any inconvenience caused.

**Fig. 1:**
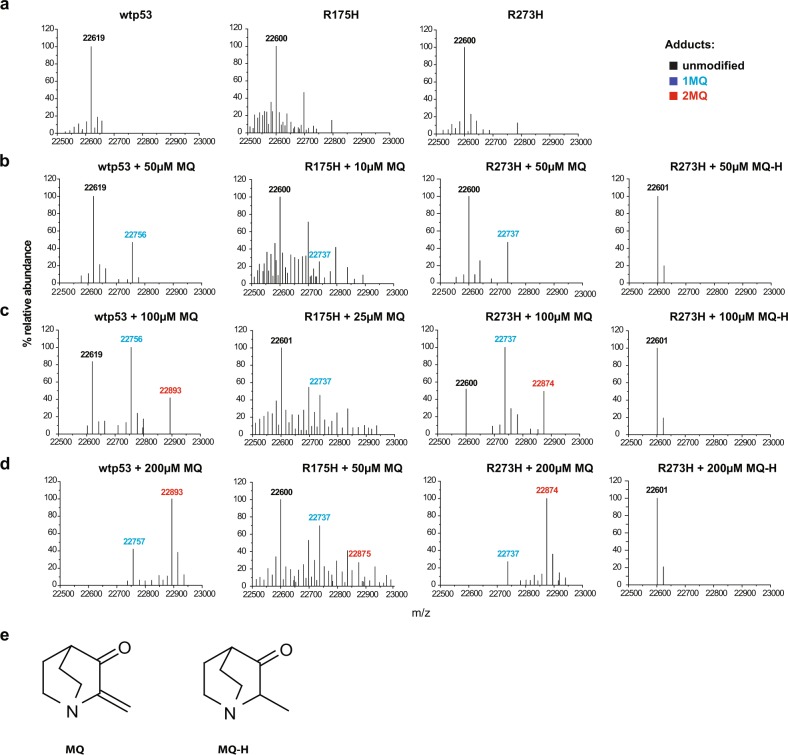
Mass measurement of wild-type, R273H and R175H p53 core domains by LTQ-MS. **a** mass spectra of p53 core domains. **b**–**d** Reaction titration with MQ or MQ-H. p53 core domains were incubated with MQ at 50–200 µM (wt and R273H) or 10–50 µM (R175H) concentration ranges. One MQ adduct increased the molecular mass of p53 core domains by 137 Da. **e** Structure of MQ and MQ-H

