# Supplementary figures and images for: APR-246 reactivates mutant p53 by targeting cysteines 124 and 277
Source: Cell Death Dis. 2018 Apr 18;9(5):439. doi: 10.1038/s41419-018-0463-7 (PMC5906465; doi:10.1038/s41419-018-0463-7)

PAb1620

DAPI

FL393

HCT116 wtp53  
+Doxorubicin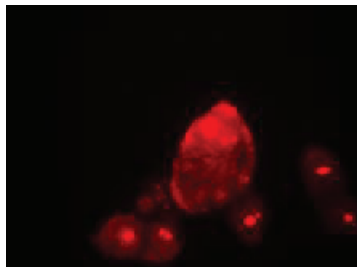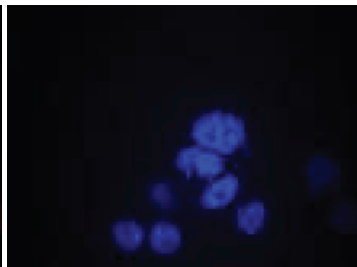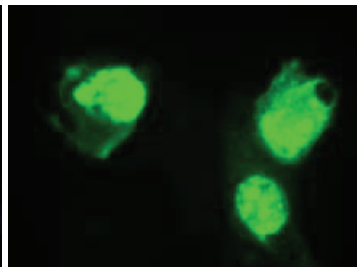

H1299-R175H

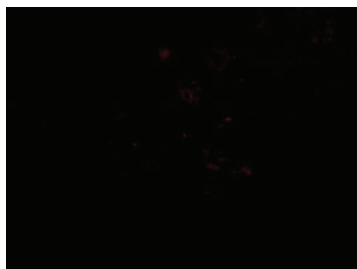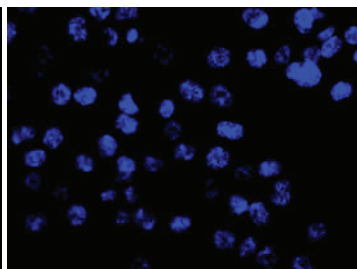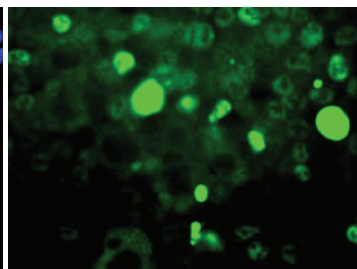H1299-R175H  
+ APR246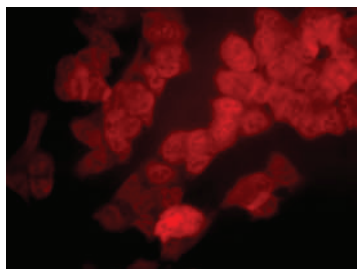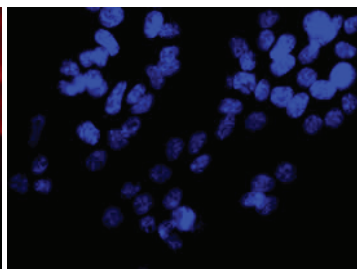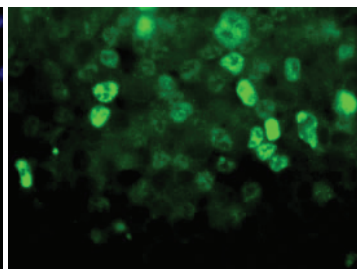

Saos-2-R273H

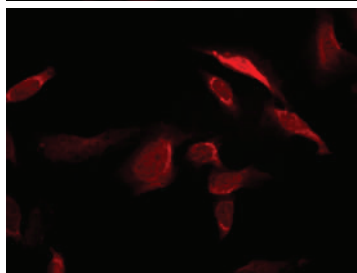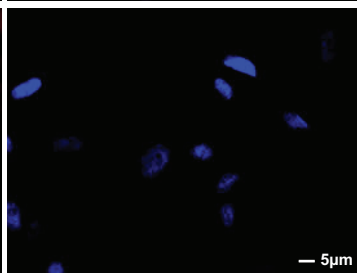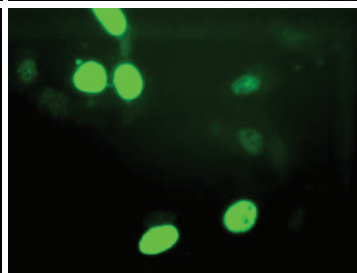

— 5μm

Supplement: Supplementary file 1 — Supplementary Figure 1(PDF 2454 kb) [file 41419_2018_463_MOESM1_ESM.pdf]

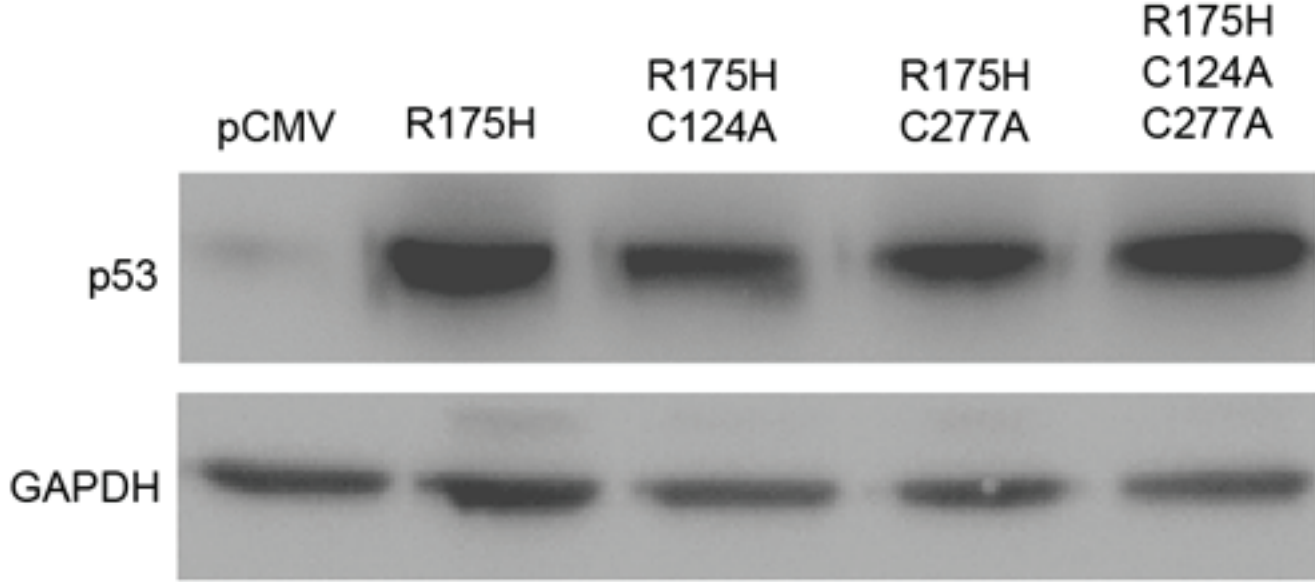

Supplement: Supplementary file 2 — Supplementary Figure 2(PDF 427 kb) [file 41419_2018_463_MOESM2_ESM.pdf]

**a**

### Annexin V

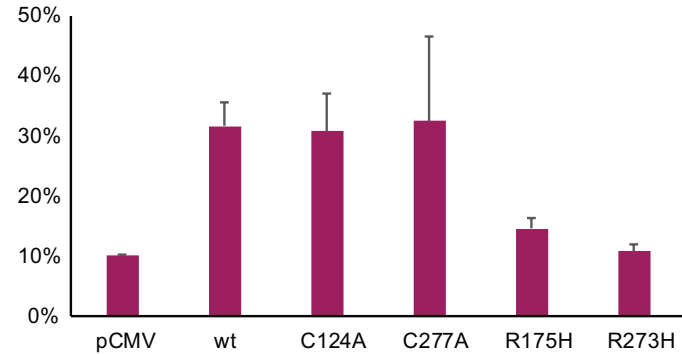**b**

### p21

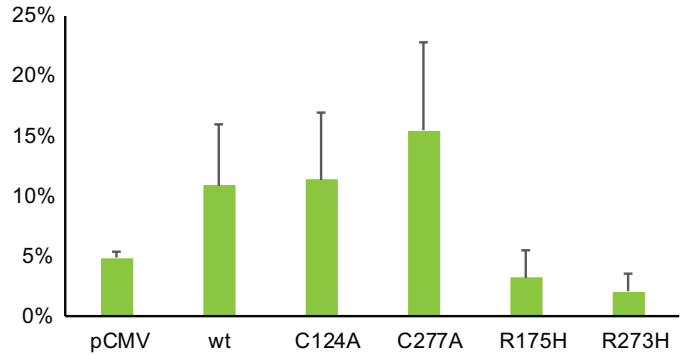

Supplement: Supplementary file 3 — Supplementary Figure 3(PDF 7 kb) [file 41419_2018_463_MOESM3_ESM.pdf]
