# Supplementary material for: APR-246 reactivates mutant p53 by targeting cysteines 124 and 277
Source: Cell Death Dis. 2018 Apr 18;9(5):439. doi: 10.1038/s41419-018-0463-7 (PMC5906465; doi:10.1038/s41419-018-0463-7)
Supplement: Supplementary file 4 — Supplementary information(DOCX 16 kb) [file 41419_2018_463_MOESM4_ESM.docx]

**Supplementary information**

**Supplementary Figure 1. The PAb1620 antibody recognizes correctly folded p53 in immunofluorescence staining.** HCT116 cells treated with Doxorubicin for 24 hours, H1299-R175H treated with APR-246 for 24 hours and untreated Saos-2-R273H cells were immunostained with the wild-type p53 conformation-specific antibody PAb1620. In parallel, cells were stained with the general p53 antibody FL-393 to show the level of p53 expression.

**Supplementary Figure 2. p53 expression levels in transiently transfected H1299 cells.**Cells were transfected with the indicated constructs and harvested 24 hours after transfection. p53 was visualized by Western blotting with p53 antibody DO-1. GAPDH was used as a loading control. The blot was first probed with DO-1 and the reprobed with GAPDH antibody. The figure shows cropped images of the whole blot.

**Supplementary Figure 3. p53 function is not abrogated by the C124A and C277A substitutions.** H1299 cells were transfected with the indicated p53 constructs and harvested after 24 hours. Annexin V induction (a) and p21 expression (b) were assessed by flow cytometry.
